# Metabolic Syndrome Features Presenting in Early Childhood in Alström Syndrome: A Case Report

**DOI:** 10.4274/jcrpe.v1i6.278

**Published:** 2010-12-08

**Authors:** Özgür Pirgon, Mehmet Emre Atabek, İlhan Asya Tanju

**Affiliations:** 1 Department of Pediatric Endocrinology, Konya Research Hospital, Konya, Turkey; 2 Department of Pediatric Endocrinology, Medical Faculty, Selçuk University, Konya, Turkey; 3 Department of Pediatrics, GATA Medical Faculty, İstanbul, Turkey; +90 332 323 67 09/5300ozpirgon@hotmail.comKonya Eğitim ve Araştırma Hastanesi, Çocuk Endokrinolojisi Polikliniği, Meram Yeni Yol Üzeri, Konya

**Keywords:** metabolic syndrome, Alström syndrome, child

## Abstract

Alström syndrome is a rare autosomal recessive disorder characterized by retinal degeneration, sensorineural hearing loss, early-onset obesity, and non-insulin-dependent diabetes mellitus. Affected individuals have normal birth weight, but growth deceleration starts at about 8-10 years of age. In patients with the disorder linked to chromosome 2, the increase in body mass index is very high in childhood and continues high thereafter. In this paper, we report a patient who had the proposed diagnostic criteria for Alström syndrome associated with metabolic syndrome starting at age 7, a relatively early age.

**Conflict of interest:**None declared.

## INTRODUCTION

Alström syndrome is a rare autosomal recessive disorder first described in 1959 (1). A wide range of clinical variability is observed among individuals with Alström syndrome, including among siblings. The major clinical features of Alström syndrome are cone-rod dystrophy resulting in childhood blindness; truncal obesity that manifests during the first year of life; insulin-resistant type 2 diabetes mellitus (T2DM); progressive sensorineural hearing loss; and infantile- or adolescent-onset dilated cardiomyopathy (2). The total number of reported cases of Alström syndrome is approximately 300. The gene mutated in Alström syndrome patients, ALMS1, has recently been identified (3, 4). The diagnosis of Alström syndrome is very difficult in early ages, since the typical morphological stigmata become evident only after the age of 6-8 years.

The clinical diagnosis of metabolic syndrome is based on the presence of an abnormal glucose metabolism, hypertension, hyperlipidemia and obesity (5). The lipid profiles of patients with metabolic syndrome are often characterized by hypertriglyceridaemia and high LDL cholesterol, and low HDL cholesterol. Patients with these abnormalities have an increased risk of premature coronary artery disease (6). We report a 7-year-old girl with Alström syndrome presenting with the features of metabolic syndrome in the pediatric age group.

## CASE REPORT

A 7-year-old female was admitted to our department due to obesity. The second child of consanguineous parents, she was born after 40 weeks of gestation with a birth weight of 3000 g. No problems were observed during the neonatal period. She was reported to have no mental retardation or any signs of motor delay.

**Figure 1:**
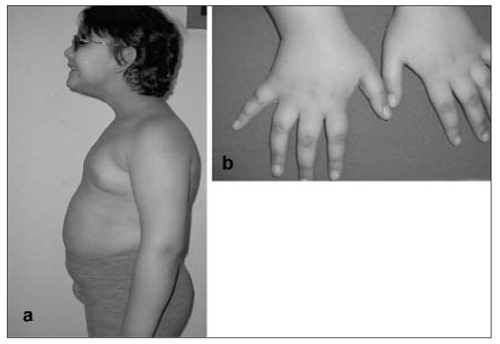
(a) Facial appearance of the patient with truncal obesity, (b) Dark shiny patches (acanthosis nigricans) on fingers

The patient weighed 32 kg (>95^th^ percentile), her height was 118.5 centimeters (25-50^th^ percentile) and head circumference was 49.5 centimeters (25^th^ percentile). Body mass index was calculated as 23 kg/m2 (>95^th^ percentile). Systolic and diastolic blood pressure values were measured as 110 mmHg and 80 mmHg, respectively. Acanthosis nigricans was noted in the axillary region and fingers, bilaterally (Figure 1). She had photophobia, and cone-rod dystrophy was detected by fundoscopic examination.

Routine investigations, including complete blood count, liver function tests, renal function tests and sedimentation rate were within normal ranges. Metabolic screening and thyroid function tests were normal. Cushing’s syndrome was excluded by dexamethasone suppression tests. Following oral administration of glucose (1.75 g glucose/kg), blood glucose and plasma insulin levels were 159 mg/dL and 152.86 mU/ml at 30 min, and 145 mg/dL and 58.97 mU/mL at 120 min, respectively (Table 1). HbA1c was 6.6%. The patient was hyperlipidemic with a total cholesterol level of 238 mg/dL and a LDL cholesterol level of 163 mg/dL. HDL cholesterol level was low (33 mg/dL). Blood uric acid level was 5 mg/dL. After metformin treatment (850 mg/day) for 6 months, the oral glucose tolerance test showed improved glucose and insulin levels (Table 1). 

Karyotype analysis with standard techniques revealed a 46,XX karyotype. The patient was diagnosed as a case of Alström syndrome clinically. Her total genomic DNA isolated from peripheral blood leukocytes was analyzed at Jackson Laboratory (Bar Harbor, ME; GB. Collin and PM. Nishina). A cytogenetic study was performed on the peripheral blood lymphocytes of the patient using high resolution and conventional banded-chromosome analysis including a molecular analysis of ALMS1 gene (GeneBank accession number AJ417593). As most of the reported mutations are harbored within exons 10 and 16, these two exons were analyzed. A silent polymorphism was detected at position 8506 (G>T E>X), a finding consistent with the clinical diagnosis of Alström syndrome. 

**Table 1:**
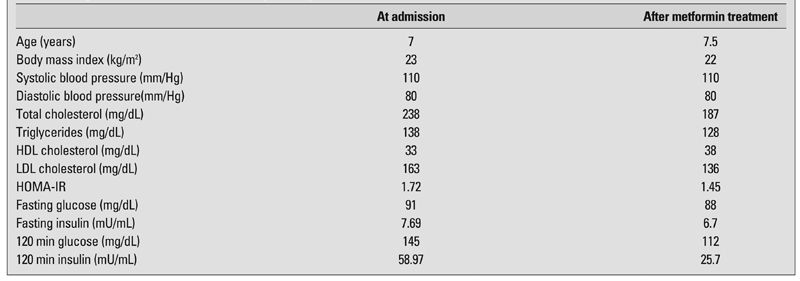
Oral glucose tolerance test results and plasma lipid levels before and after metformin treatment

## DISCUSSION

In childhood, most individuals with Alström syndrome have hyperinsulinemia and associated acanthosis nigricans, hypertriglyceridemia, accelerated skeletal maturity (resulting in short adult stature), scoliosis or kyphosis, and low growth hormone levels. With increasing age, affected individuals may develop T2DM, other metabolic disturbances, and renal failure (2). The diabetes mellitus in Alström syndrome is the result of tissue resistance to the actions of insulin, as demonstrated by elevated plasma insulin levels and glucose intolerance, findings often present in childhood. In the literature, diabetes was diagnosed between 15 and 25 years of age in the majority of patients, with hyperinsulinemia often preceding the diabetes (7). 

Our patient with Alström syndrome showed symptoms of metabolic syndrome including obesity, dyslipidaemia and glucose intolerance at age 7 years. Currently, there is no reported evidence of excessive atherosclerotic disease and premature coronary artery disease in Alström syndrome patients. However, in a recent report, 4 of 22 of Alström syndrome patients were reported to have succumbed to cardiac causes at 26 days, 2 years, 6 years, and 19 years of age. In addition to features of Alström syndrome, the metabolic syndrome may lead to complications, such as hypertension, hypertriglyceridemia, hyperuricemia, hyperfibrinogenemia, and impaired glucose tolerance or diabetes, which are all risk factors for premature atherosclerotic disease (8). In conclusion, increased vascular risk is a late complication to be added to the other typical features of Alström syndrome, as suggested for Bardet-Biedl syndrome and Cohen syndrome (9, 10).

In our patient, blood pressure measurements were not above the 95th percentile for age and height on two separate measurements. However, she had high lipid levels at an early age. Her lipid pattern reflected the typical dyslipidaemia (high triglycerides and total cholesterol levels) observed in overweight individuals. Despite normal fasting plasma glucose and insulin levels, the 2 hour plasma glucose concentration during an oral glucose tolerance test showed glucose intolerance with marked hyperinsulinemia. Treatment with metformin and lifestyle changes, including body weight control with diet modification, were recommended. After six months, the treatment proved to be effective in reducing insulin and lipid levels and in ameliorating the complications of metabolic syndrome. 

Alström syndrome may be particularly difficult to diagnose because it is rare and because it has a complex phenotype that evolves over time with multiple possible presentations. Our patient is another example demonstrating that diabetes or metabolic syndrome may occur in Alström syndrome in the early stages of life, and that obese children with Alström syndrome should be evaluated for metabolic syndrome. Insulin-resistant children at a younger age who can compensate by hyperinsulinemia may escape diabetes, but are still prone to other complications. Without an oral glucose tolerance test, fasting glucose and insulin measurements may fail to identify metabolic syndrome risk in children with Alström syndrome. It can also be concluded that metformin can exert some effect on hyperinsulinemia, impaired glucose tolerance, dyslipidemia and other metabolic disturbances in patients with Alström syndrome.
